# An Unusual Transudative Pleural Effusion Succeeded by Pulmonary and Brain Edema and Death

**DOI:** 10.1155/2012/896409

**Published:** 2012-03-28

**Authors:** Sayyed Gholam Reza Mortazavimoghaddam, H. R. Riasi

**Affiliations:** ^1^Pulmonary Ward, Vali-e-Asr Hospital, Birjand University of Medical Sciences, Birjand 9718766995, Iran; ^2^Neurologic Ward, Vali-e-Asr Hospital, Birjand University of Medical Sciences, Birjand 9718766995, Iran

## Abstract

Here we report a 22-year old woman with massive and bilateral transudative effusion succeeded by pulmonary edema and brain edema and death. Investigations for systemic disorders were negative. Exacerbation of dyspnea after intravenous fluid infusion was a main problem. As effusion was refractory to medical treatment, the patient was referred for surgical pleurodesis and bilateral surgical pleurodesis were done separately. Postsurgically, dyspnea exacerbation occurred after each common cold infection. Vertigo and high intracranial pressure were also a problem postsurgically. CSF pressure was 225 mm/H_2_O. Therapeutic lumbar puncture was done in two sequential weeks, and the patient was on acetazolamide 250 mg/trivise a day. Despite the medical treatment, progressive dyspnea, headache, and high intracranial pressure followed by death nine months after pleurodesis. As there is a gradient of pressure between pleura and CSF, after pleurodesis brain edema must be a consequence of inversing this gradient. In conclusion, when there are any abnormalities about fluid volume or pressure in any of these cavities, we have to study other cavities.

## 1. Introduction

Transudative pleural effusion is usually due to systemic condition and the pleural surfaces are intact per se. Among the conditions for transudative pleural effusion are congestive heart failure, cirrhosis, and nephritic syndrome [1]. Local pleural pathologies are exceedingly rare in the transudative fluid accumulation. In some cases of transudative pleural effusion, the cause of a pleural effusion may not be evident following initial investigations. Indeed effusions that are classically exudative can be transudative in some cases, particularly with malignancy and pulmonary embolism [2]. Overlay transudative pleural effusions are consequences of increased hydrostatic, decreased oncotic or abnormal anatomical defect between pleura and spaces with transudative fluid such as urine, CSF, and peritoneal dialysis fluid. However, in the absence of systemic condition such as cardiac or liver disease and also in the absence of anatomical connection between pleura and space with transudative effusion, there has not been report of such refractory massive and bilateral transudative pleural effusion. In addition, despite the role of intracranial hypertension in the genesis of neurogenic pulmonary edema [3], there has not been a report or explanation for relationship between pleural effusion and increased intracranial hypertension. Here, there is the first report of massive and bilateral transudative pleural effusion complicated by increased intracranial hypertension and several bout of pulmonary edema, succumbed the patient to death nine months after bilateral pleurodesis.

## 2. Case Presentation

**Figure 1:**
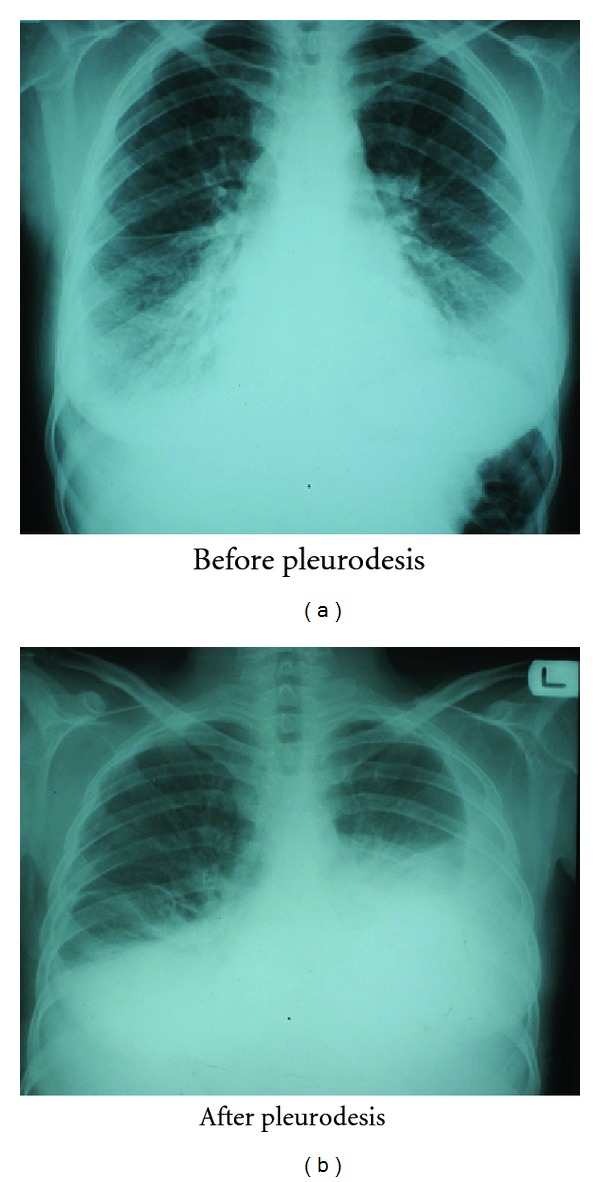
Chest X-ray of the patient before and after pleurodesis.

A 23-year old woman, whose chief complaint was dyspnea, was referred by general practitioner to pulmonologist. After initial examination and radiographic study, diagnosis of bilateral pleural effusion was ascertained (Figure 1). In her past medical history, recurrent headache, vertigo, and exacerbation of dyspnea after intravenous fluid infusion were reported. Tapped fluid from pleural cavity was sent for biochemistry, cytological, and microbiologic studies.

The result showed that she had transudative pleural effusion. With complete studies of cardiovascular, urinary, hepatic, blood, and endocrine systems, no disorder has been found as the cause of pleural effusion. Despite the transudative fluid, pleural needle biopsy was taken in hope of clarification; however, there were also no specific pathologic changes. Because of sever dyspnea on day after pleural biopsy, and massive effusion on the left, a left side chest tube was inserted. A large volume of effusion was drained. An unusual presentation was drainage of more than 800 mL/day of pleura fluid on succeeding days. As volume of fluid drainage was high on sequencing days, the chest tube remained in place and the patient referred for surgical pleurodesis. Bilateral surgical pleurodesis was tacked on two separate times and the patient was in fairly acceptable condition on the succeeding months, except for dyspnea exacerbation after each commune cold infection or IV fluid infusion. She had increasing headache, transient darkness in visual field, transient diplopia and vertigo in the last presentation, nine months after bilateral pleurodesis. Bilateral papillary edema was confirmed by neurological examination and the results of diagnostic approach including brain MRI were normal. Lumbar puncture showed completely normal biochemistry, cytopathology, and serologic VDRL test. CSF pressure was 225 mm/H_2_O. During two weeks of hospitalization, 35 CC of CSF has been punctured each week, and after partial relieving of symptom and decreasing of papilledema, she was discharged. Acetazolamide 250 mg three times daily has been prescribed as medical treatment for lowering CSF pressure. She is visited each month by pulmonologist and neurologist. Progressive dyspnea, vertigo, headache and refractory brain and pulmonary edema made the patient fall into respiratory failure, unconsciousness, and finally death.

## 3. Discussion

The feature unique to our case is massive and bilateral transudative pleural effusion characterized by persistent, intractable, and rapid accumulation after each tap. In addition, high daily drainage during chest tube insertion, several bouts of pulmonary edema, and cerebral edema after bilateral pleurodesis were problematic for our patient.

Persistent and rapid accumulation of pleural effusion is a usual presentation in patient with malignancy, cirrhosis, and cardiac failure [4]. We review otherwise the PubMed literature in English and encountered a lot of symptomatic transudative pleural effusion after spinal surgery, thoracic surgery and/or after durra shunting for pseudo tumor cerebri [2–5]. All of these cases resulted from an anatomical communication between durra and pleura. Anatomical communication may result in rapid accumulation of large effusions due to large amount of CSF production each hour and also relative positive pressure of the spinal fluid in comparison to the negative intrapleural pressure [6].

However, in the present case there was no evidence of malignancy or any functional or anatomical disturbance of heart, kidney, liver, or abnormal anatomical communication between CSF and pleural space.

Over the extensive investigations, it did not result in exploring the cause or causes of this intractable pleural effusion. It may be an undetermined etiology of transudative pleural effusion. However, this is contrary to the subject of undetermined etiology of pleural effusion, as most studies about undetermined form are about exudative pleural effusion [2, 5].

Indeed in the present case, despite transudative effusion, we were in inescapable to consider bilateral surgical pleurodesis because of severely symptomatic patient. Several types of refractory pleural effusion have been successfully managed by pleurodesis, including those due to chronic ambulatory peritoneal dialysis [7], yellow nail syndrome [8], and heart failure [6]. However, a Medline search confirmed that the occurrence of an increased intracranial pressure following pleurodesis has not been reported before. As pleural effusion was controlled, several bouts of pulmonary edema and gradual increased intracranial pressure came to problematic conditions in our patient.

Regarding the absence of anatomical correlation between durra and pleura in our case, we suppose a systemic condition such as increased vascular permeability or out flow reduction as the underlying pathophysiologic mechanism common to increased intracranial hypertension and pulmonary edema and pleural effusion. In this regard, the role of intracranial hypertension in the genesis of neurogenic pulmonary edema is a good example. When high intracranial pressure occurs, the result in lung is changing pulmonary circuit and doubling of lymphatic flow [9].

Thus, considering a possible relationship between increased vascular permeability, decreased drainage or both and body space fluid accumulation or interstitail edema raises further questions.

First, there may be a considerable numbers of patients who are under treatment only for pleural effusion, but have rising intracranial pressure at the same time or vice versa and unfortunately the second situation is not diagnosed or diagnosed when patients are affected by irreversible sequels. We suggest that each patient whose chief complaint is transudative pleural effusion even with known etiology must be carefully examined for sign of pseudo tumor cerebri. On the other hand, each patient whose chief compliant is rise intracranial pressure (ICP), careful chest examination is necessary and chest X ray for pleural effusion and interstitial edema should be obtained.

Second, some signaling may appear to play a critical role in the maintenance or disturbances of vascular integrity and regulation of basal permeability. These factors may influence from conditions such as malignancy, intracranial hypertension, or congenital defects. We were not interested in these factors at the time, but it will be important to consider more investigation on serum and serosal fluid sample about vasoactive factors such as VEGF and FGF and gen polymorphism in such patients.

Ultimately transudative pleural effusion might be consequences of another but very rare systemic condition labeled as endogenous high permeability membrane or systemic decreased of fluid drainage. We suggest the most sophisticated study of fluid accumulation mechanisms in patient with idiopathic transudative pleural effusion or increased intracranial pressure.
